# Gender Affirming Surgery During Canadian Plastic Surgery Residency: A Survey of Plastic and Reconstructive Surgery Residents’ Experiences

**DOI:** 10.1177/22925503261471514

**Published:** 2026-08-03

**Authors:** Camille Zeitouni, Alexandra Speak, Frédérique Leroux, Michelle Bonapace-Potvin, Dominique Tremblay

**Affiliations:** 1 Division of Plastic and Reconstructive Surgery, 3688 Dalhousie University, Halifax, NS, Canada; 2 Division of Plastic and Reconstructive Surgery, 12368 Université de Montréal, Montréal, QC, Canada; 3 Division of Plastic and Reconstructive Surgery, 5635 University of Minnesota, Minneapolis, MN, USA

**Keywords:** gender affirming care, gender affirming surgery, plastic surgery residency, surgical education, soins d’affirmation de genre, chirurgie d’affirmation de genre, résidence en chirurgie plastique et reconstructrice, formation chirurgicale

## Abstract

**Introduction:**

Gender affirming surgery (GAS) is an umbrella term for procedures that can be a part of the transition process for transgender and nonbinary individuals. Plastic surgeons play an important role in gender affirming surgical care. Our purpose was to assess the GAS landscape in Plastic and Reconstructive Surgery (PRS) residency programs across Canada, including both the didactic and clinical context, along with assessing PRS residents on their perceived interest and competence relating to performing GAS.

**Methods:**

A survey was created based on existing literature relating to gender affirming care in surgical and medical postgraduate education. The survey was distributed to all PRS residents in Canada through their program director. Results were analysed using descriptive statistics.

**Results:**

Almost all residents surveyed agreed that transgender care is an important part of PRS. Despite this, little educational content regarding GAS was seen. The majority of individuals had never been exposed to any GAS clinically despite having at least one surgeon who performs GAS affiliated with their institution. For individuals who have had exposure, top surgery was the most common procedure seen. While few residents plan on pursuing a fellowship in GAS, some residents did comment they planned to offer top surgery or facial feminization as general plastic surgeons.

**Conclusion:**

The majority of PRS residents demonstrated a positive response towards the importance of GAS in PRS. As access to GAS in Canada remains limited, providing greater exposure to GAS in PRS residency may help in promoting willingness and competence to perform these procedures.

## Résumé

**Introduction:**

La chirurgie d’affirmation de genre (CAG) est un terme générique désignant les interventions pouvant s’inscrire dans le processus de transition des personnes transgenres et non binaires. Les chirurgiens plasticiens jouent un rôle important dans la prise en charge chirurgicale de l’affirmation de genre. Notre objectif était d’évaluer le contexte actuel de la chirurgie d’affirmation de genre dans les programmes de formation en chirurgie plastique et reconstructive (CPR) à travers le Canada, tant sur le plan théorique que clinique, ainsi que d’évaluer l’intérêt et les compétences perçus par les résidents en CPR concernant la pratique de la chirurgie d’affirmation de genre.

**Méthodes:**

Un questionnaire a été élaboré à partir de la littérature existante relative aux soins aux personnes transgenres dans la formation postuniversitaire en chirurgie et en médecine. Ce questionnaire a été distribué à tous les résidents en CPR du Canada par l’intermédiaire de leur directeur de programme. Les résultats ont été analysés à l’aide de statistiques descriptives.

**Résultats:**

La quasi-totalité des résidents interrogés s’accordaient à dire que les soins aux personnes transgenres constituent un volet important de la CPR. Malgré cela, le contenu pédagogique consacré à la CAG s’est avéré très limité. La majorité des personnes n’avaient jamais été exposées à la chirurgie d’affirmation de genre en milieu clinique, bien qu’au moins un chirurgien pratiquant la CAG soit affilié à leur établissement. Parmi les personnes ayant eu une exposition à cette pratique, la chirurgie du torse était l’intervention la plus couramment observée. Si peu de résidents envisagent de suivre une formation spécialisée en CAG, certains ont toutefois indiqué qu’ils prévoyaient de proposer la chirurgie du torse ou la féminisation du visage en tant que chirurgiens plasticiens généralistes.

**Conclusion:**

La majorité des résidents en chirurgie plastique et reconstructrice ont manifesté une attitude positive quant à l’importance de la CAG dans leur formation. L’accès à la CAG restant limité au Canada, une plus grande exposition à ce type de chirurgie au cours de la résidence en chirurgie plastique et reconstructrice pourrait contribuer à renforcer la volonté et les compétences nécessaires à la réalisation de ces interventions.

## Introduction

Gender affirming surgery (GAS) encompasses a range of surgical procedures that may form part of the transition process for transgender and nonbinary individuals to better reflect their gender identity. Within plastic and reconstructive surgery (PRS), this includes procedures such as mastectomy, breast augmentation, vaginoplasty, phalloplasty, metoidioplasty and facial gender affirmation surgery. Access to GAS remains limited in many regions in Canada, as only a small number of surgeons currently perform these operations, and many centers face year-long wait times.^1^ These prolonged delays have been associated with worsened mental health outcomes and are implicated as contributing factors in the elevated rates of suicidal ideation and suicide attempts reported among transgender Ontarians.^2^

A significant contributor to the limited access to gender affirming care may be the deficiency of transgender-related curricula within both undergraduate and postgraduate medical education.^3–5^ Previous research has shown substantial gaps in trainee exposure to transgender health across multiple specialties.^3,6^ For example, over 30% of urology and plastic surgery programs in the United States report no clinical exposure to transgender patients.^7^ A previous Canadian survey showed only 50% of endocrinology, 35% of psychiatry, 10% of family medicine, and 0% of urology residents feel confident providing gender affirming care.^3,5,6^ These findings highlight a broader educational deficiency that may perpetuate disparities in access to care.

The demand for GAS continues to rise, underscoring the need to train future plastic surgeons who are both competent and comfortable providing gender affirming procedures. To date, no studies have evaluated the transgender-related content or GAS exposure within Canadian plastic surgery residency programs. The objective of this study was to assess the current state of gender affirming surgical education among Canadian plastic surgery residents by examining: (1) the presence of GAS within academic curricula (including journal clubs, lectures, grand rounds, patient talks, and examination); (2) the presence of GAS exposure within clinical care (in both clinic and operative settings); and (3) residents’ perceived readiness and competence to perform GAS procedures.

## Methods

### Survey Development

A survey was developed based on existing literature examining gender affirming care in surgical and medical postgraduate education.^3,7–13^ Notably, the instrument was largely adapted from the survey used by Magoon and colleagues in their study “The Current State of Gender-Affirming Surgery Training in Plastic Surgery Residency Programs as Reported by Residency Program Directors.^8^ The final version consisted of 22 items, including multiple choice, Likert-scale, and short-answer questions. These covered three main domains: (1) demographic characteristics of respondents, (2) exposure to GAS during residency training, and (3) resident attitudes and perceptions regarding GAS. Exposure was defined as any clinical care encounter in which residents were active members of the care team, including participation in outpatient clinical visits and operative procedures in the operating room. A copy of the complete survey is available in the Supplemental Materials.

### Survey Distribution and Participants

The survey was distributed electronically to all current plastic surgery residents across Canada through their respective program directors. Responses were collected using Google Forms from June 2023 to June 2024. Participation was voluntary and anonymous, and informed consent was obtained from all individuals prior to survey completion.

### Statistical Analysis

SPSS software was used for statistical analysis and basic survey-appropriate descriptive statistics were calculated to report results.

## Results

### Response Rate and Demographics

**Figure 1.**
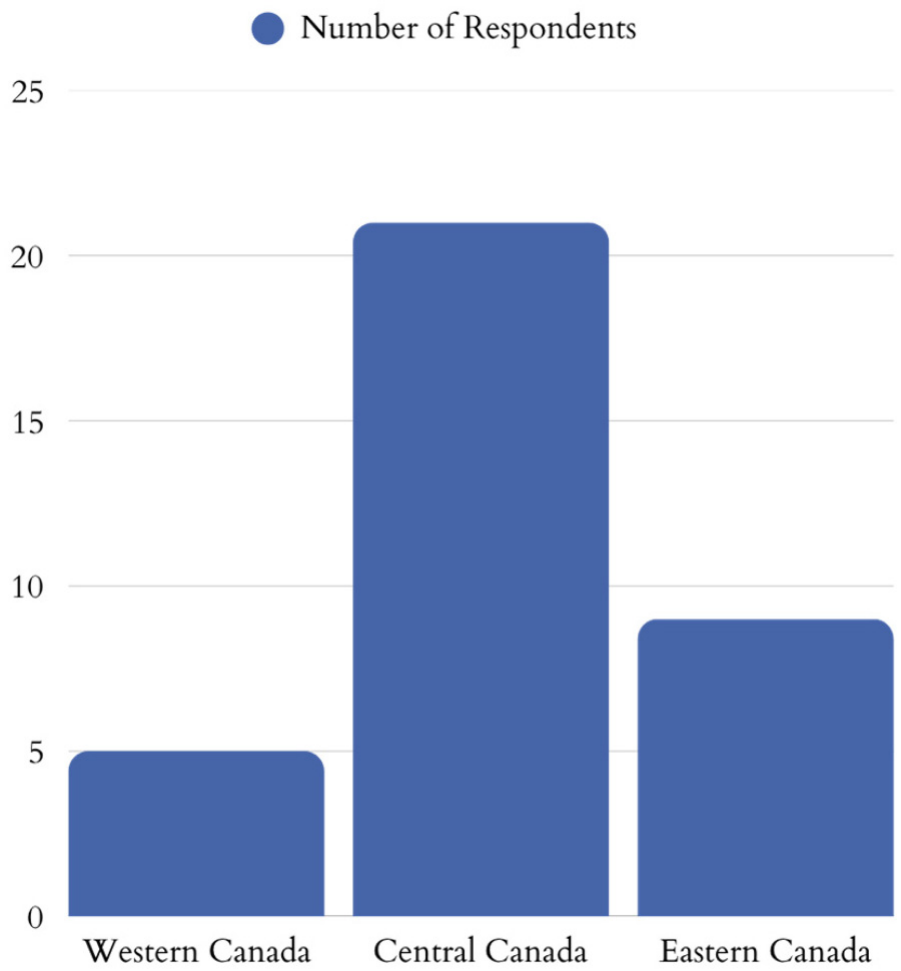
Distribution of survey respondents by training region.

A total of 36 residents responded to the survey, representing an approximate response rate of 25%. Respondents were evenly distributed across all levels of training (R1–R5), with a 1:1 male-to-female ratio. Respondents ranged in age from 24 to 36 years. Most Canadian plastic surgery programs were represented, except for McMaster University and the University of British Columbia. Figure 1 shows the respondents by region.

### Attitudes Towards Gender Affirming Care

The majority of residents expressed strong support for the inclusion of GAS within plastic surgery training. Overall, 94% of respondents agreed or strongly agreed that caring for transgender patients is an important component of both the specialty and residency education (Table 1). More than 70% of residents reported feeling comfortable asking patients for their pronouns.

**Table 1.** Attitudes Towards GAS.

Statement	Strongly Agree/Agree
Care for transgender patients is an important part of the plastic and reconstructive specialty.	94%
It is important for GAS to be included within your training as a plastic and reconstructive surgeon.	94%
It is important that plastic surgery residents have exposure to patients receiving bottom/genital surgery.	81%
It is important that plastic surgery residents have exposure to patients receiving top surgery, including mastectomy and breast augmentation.	94%
It is important that plastic surgery residents have exposure to patients receiving facial surgery	86%

### Didactic Exposure

With regard to formal teaching, residents reported an average of 2.2 h of lectures or seminars, 1.3 h of journal clubs or grand rounds, and 20 min of patient talks per year devoted to GAS-related topics. More than 60% of respondents wished for additional theoretical exposure to gender affirming care within their residency curriculum.

### Clinical Exposure

**Figure 2.**
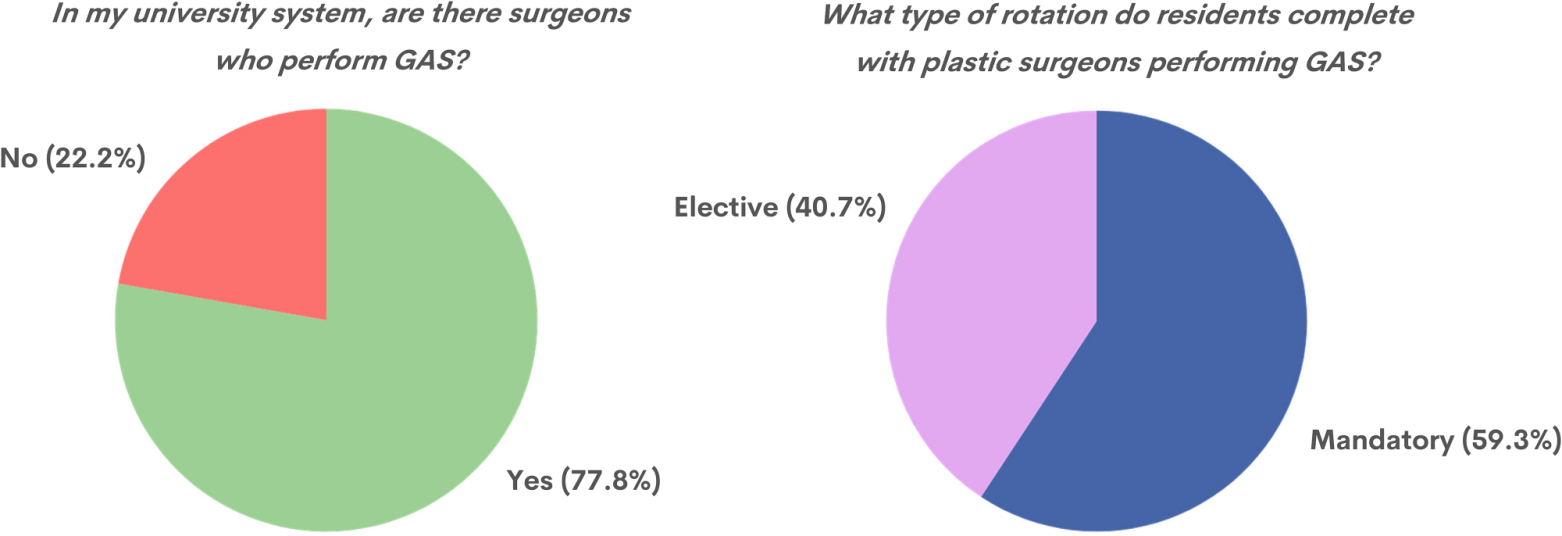
Exposure to clinical GAS.

Over 75% of respondents indicated that at least one surgeon within their university system performs gender affirming procedures. Among these, 44% reported having a mandatory rotation, while 30.6% indicated that GAS rotations were available as electives (Figure 2).

**Figure 3.**
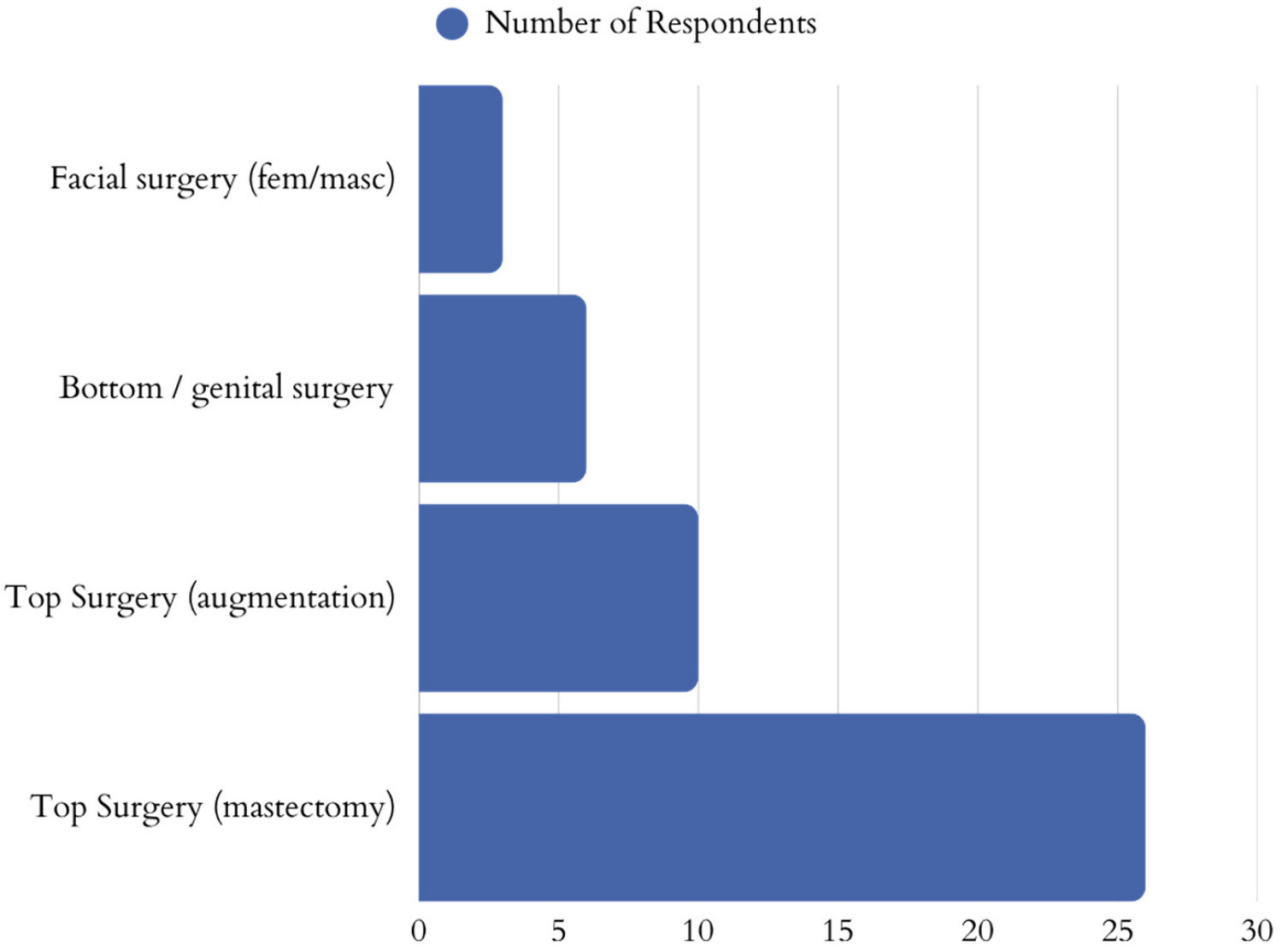
GAS Procedures encountered by Canadian plastic surgery residents.

**Figure 4.**
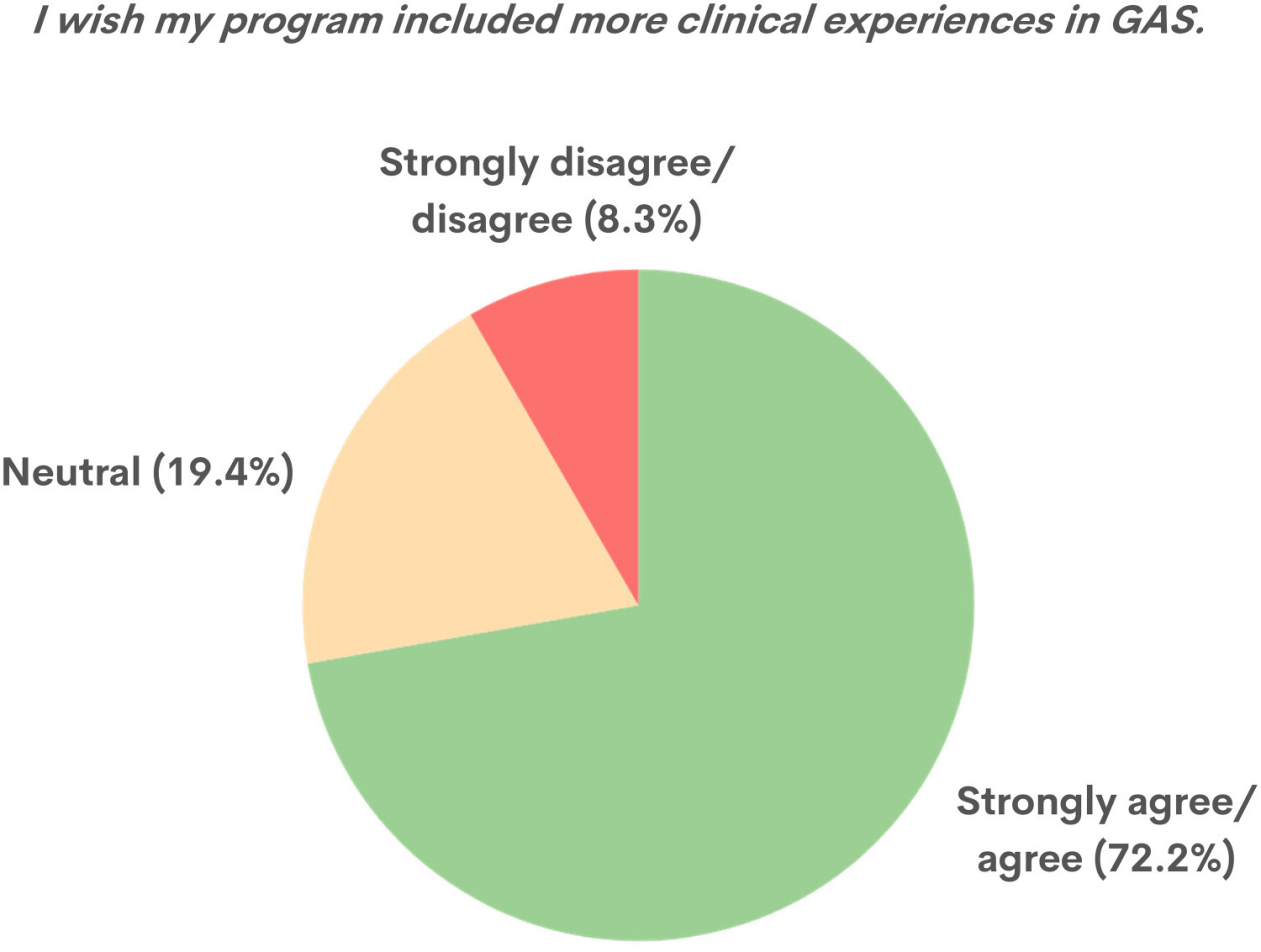
Resident attitudes toward clinical exposure to GAS.

Top surgery was the most frequently encountered procedure, with residents reporting an average of 12 cases over five years of training (Figure 3). Of the six residents who had exposure to genital (“bottom”) surgery, three did so through elective rotations in the United States. Only three residents (10.7%) reported having any exposure to facial feminization or masculinization surgery. The majority (>70%) wished for more clinical exposure to GAS (Figure 4).

### Interest and Preparedness

**Figure 5.**
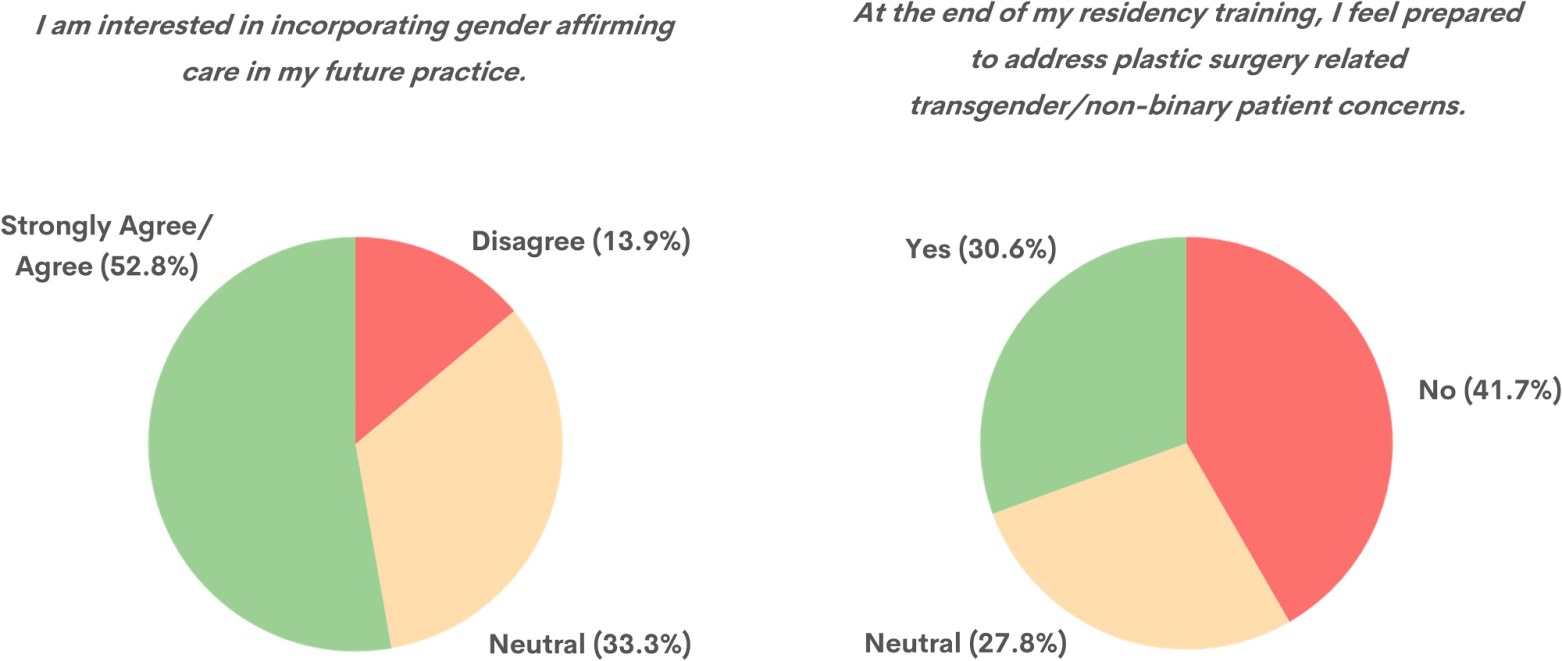
Resident preparedness and interest in performing GAS post-residency.

**Figure 6.**
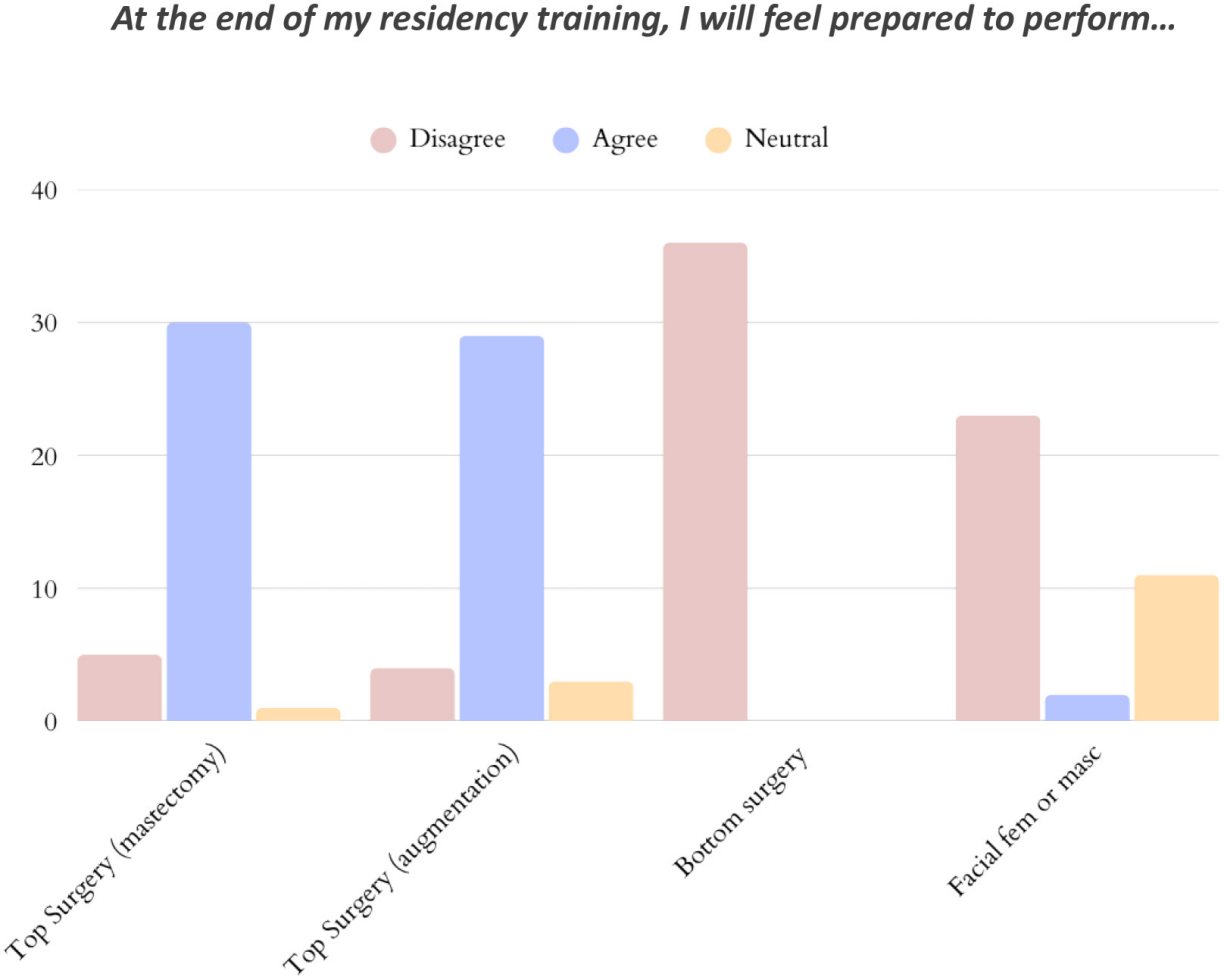
Preparedness to perform GAS post-Canadian plastic surgery residency.

More than half of residents expressed interest in incorporating gender affirming care into their future practice. However, only 30% felt prepared to address plastic surgery-related transgender concerns at the completion of residency training (Figure 5). Self-reported preparedness to perform specific GAS procedures is summarized in Figure 6.

## Discussion

GAS represents a rapidly expanding domain within PRS. The number of GAS procedures performed globally continues to increase, reflecting the growing recognition of the need for gender affirming care as an integral part of comprehensive surgical practice.^10^ In this context, an important question arises: are Canadian plastic surgery residents receiving sufficient exposure and training to competently provide these procedures or manage associated postoperative complications?

Our study represents the first national assessment of GAS exposure and education among Canadian plastic surgery residents. The findings highlight an encouragingly positive attitude among residents towards gender affirming care, with the majority expressing interest in incorporating elements of GAS into their future practice. However, both didactic and clinical exposures to GAS remain limited, with most residents reporting only around two hours of formal teaching per year and minimal hands-on experience, particularly beyond top surgery. Our findings suggest the current residency curricula may not fully adequately prepare graduates to meet the growing demand for gender affirming surgical care in Canada.

Exposure to GAS during residency appears to be closely tied to the regional availability of surgical services. Residents training in Montreal benefit from proximity to the GRS Montreal, Canada's largest center for gender affirming procedures, which offers extensive and varied clinical exposure (on average over 100 gender affirming cases per month).^14^

Similar opportunities exist in larger cities such as Toronto and Vancouver, where a broader range of GAS procedures are performed. In contrast, provinces such as Nova Scotia have local surgeons who perform top surgery, but patients seeking genital “bottom” surgery must travel to Quebec, where these procedures are still covered under Nova Scotia's provincial health insurer (MSI).^15^ Although uncommon, postoperative complications in these patients traveling out-of-province occasionally present to emergency departments, requiring local management. These regional disparities highlight the importance of ensuring that all Canadian plastic surgery residents, regardless of training location, develop foundational competence in the management of GAS patients, even if they do not perform these procedures themselves.

The training gaps identified in this Canadian plastic surgery study mirror findings across US plastic surgery, obstetrics and gynaecology, and urology programs. A 2023 systematic review across six surgical specialties across the US and Canada found that less than half of trainees received any gender affirming care training, and less than half of program directors reported offering such training.^16^ Moreover, geographic disparities highlighted in this study also seem to be consistent across specialties, as US urology residents in Western (74%) and North Central (72%) regions reported significantly more exposure to GAS than other regions (*p* ≤ .01).^9^ Finally, only 51% of American obstetrics and gynaecology programs offered any transgender health education despite the American College of Obstetricians and Gynaecologists’ (ACOG) recommendations.^17,18^

A notable limitation of our study is the absence of responses from two major Canadian plastic surgery programs: The University of British Columbia (UBC) and McMaster University. This omission is particularly significant given that UBC houses an active GAS program, representing a major source of clinical and educational exposure for residents in Western Canada. The regional skew in our participant pool towards Quebec and Atlantic Canada likely reflects the training locations of the study authors. This, in turn, limits the generalizability of our findings across the country. Additionally, as with all survey-based studies, self-selection and response bias remain potential limitations, as residents with stronger opinions or interests in GAS may have been more likely to participate.

Despite these limitations, our study underscores a clear demand among Canadian plastic surgery residents for more structured exposure to GAS. To address the discrepancy between current residency exposure and the increasing demand for GAS, a national approach may be warranted. One potential strategy could involve the establishment of a mandatory pan-Canadian rotation in GAS, integrated into the core curriculum of plastic surgery programs. This would ensure that all Canadian residents, regardless of their training institution, are exposed to the intricacies of gender affirming care. Another approach could include the formal creation of gender-affirming-specific Entrustable Professional Activities (EPAs) within the competency-based medical education (CBME) framework.

However, the feasibility of such an initiative would need to account for several logistical challenges, including the limited number of high-volume training sites, funding and travel constraints, and alignment with existing CBME frameworks. Addressing these barriers would be essential to ensure equitable access and sustainable implementation across programs.

## Conclusion

In conclusion, addressing these gaps through curriculum development and national coordination would enhance not only surgical competency while improving access and quality of care for transgender and nonbinary patients. Future work should explore strategies to integrate GAS training into core residency curricula and assess the impact of such interventions on both educational outcomes and patient care delivery.

## Supplemental Material

sj-docx-1-psg-10.1177_22925503261471514 - Supplemental material for Gender Affirming Surgery During Canadian Plastic Surgery Residency: A Survey of Plastic and Reconstructive Surgery Residents’ Experiences Supplemental material, sj-docx-1-psg-10.1177_22925503261471514 for Gender Affirming Surgery During Canadian Plastic Surgery Residency: A Survey of Plastic and Reconstructive Surgery Residents’ Experiences by Camille Zeitouni, Alexandra Speak, Frédérique Leroux, Michelle Bonapace-Potvin and Dominique Tremblay in Plastic Surgery
