# Supplementary material for: Gender Affirming Surgery During Canadian Plastic Surgery Residency: A Survey of Plastic and Reconstructive Surgery Residents’ Experiences
Source: Plast Surg (Oakv). 2026 Aug 3:22925503261471514. Online ahead of print. doi: 10.1177/22925503261471514 (PMC13433571; doi:10.1177/22925503261471514)
Supplement: Supplementary file 1 [file sj-docx-1-psg-10.1177_22925503261471514.docx]

**Survey – Trans Care in Plastic Surgery Residency**

This surgery aims to investigate how Plastic and Reconstructive Surgery residency in Canada are incorporating trans care and gender affirming surgery (GAS) within training and assessing resident’s attitudes and preparedness towards providing GAS in the future.

Participation in this study involves responding to this survey (about 10 minutes).

**All answers are anonymous.**

If you have any questions or concerns, please contact:

Consent

I wish to participate in this study – Y/N

Demographic Data

1. Post-Graduate Year (ex: PGY1, PGY2, PGY3, PGY4, PGY5, PGY6)
2. Gender (M, F, NB, Trans, Other)
   a. If other: specify
3. Age
4. University

Assessment of GAS training within educational activities

1. On average, how many hours of **journal clubs/grand rounds** per year are devoted to training in providing GAS?
2. On average, how many hours of **classes** per year are devoted to training in providing GAS?
3. On average, how many hours of **patient talks** per year are devoted to training in providing GAS?
4. I wish my program included more educational content regarding trans care and GAS
   a. Strongly disagree
   b. Disagree
   c. Neutral
   d. Agree
   e. Strongly agree

Assessment of GAS training within clinical context

1. In my university system, there are surgeons that perform GAS? Y/N
2. If you answered “Yes” to the previous question, do plastic surgery residents rotate with surgeons who perform gender affirming surgery?
   i. Yes mandatory
   ii. Yes, if chosen for elective
   iii. No
   iv. N/A
3. Have you had exposure to patients receiving:
   a. Bottom/genital surgery (including phalloplasty both vaginoplasty) (Y/N)
      i. How many cases
   b. Top surgery (mastectomy) (Y/N)
      i. How many cases
   c. Breast augmentation (Y/N)
      i. How many cases
   d. Facial surgery (feminization or masculinization) (Y/N)
      i. How many cases
   e. Other gender affirming procedures: (Y/N)
      i. Name the procedure(s)
      ii. How many cases
4. I wish my program included more clinical experiences in trans care and GAS
   a. Strongly disagree
   b. Disagree
   c. Neutral
   d. Agree
   e. Strongly agree

Assessment of attitudes regarding GAS training in plastic surgery training (rate each statement using Likert scale – *example shown for first question*)

1. Care for transgender patients is an important part of the plastic and reconstructive specialty.
   a. Strongly disagree
   b. Disagree
   c. Neutral
   d. Agree
   e. Strongly agree
2. It is important for GAS be included within your training as a plastic and reconstructive surgeon.
3. It is important that plastic surgery residents have exposure to patients receiving bottom/genital surgery.
4. It is important that plastic surgery residents have exposure to patients receiving top surgery, including mastectomy and breast augmentation.
5. It is important that plastic surgery residents have exposure to patients receiving facial surgery.
6. It is important for plastic surgeons who perform GAS to have a fellowship training in these surgeries.

Assessment for preparedness in providing GAS after residency

1. I am comfortable asking patients about their pronouns.
   a. Strongly disagree
   b. Disagree
   c. Neutral
   d. Agree
   e. Strongly agree
2. At the end of my residency training, I will feel prepared to address plastic surgery related transgender concerns.
3. At the end of my residency training, I will feel prepared to perform:
   a. Top surgery (mastectomy)
      i. Strongly disagree
      ii. Disagree
      iii. Neutral
      iv. Agree
      v. Strongly agree
   b. Top surgery (augmentation)
   c. Bottom/genital surgery
   d. Facial surgery
4. I am interested in incorporating trans-related care in my future practice.
   a. Strongly disagree
   b. Disagree
   c. Neutral
   d. Agree
   e. Strongly agree

If you wish to provide more information about previous question "I am interested in incorporating trans-related care in my future practice" please use text box below

Is there anything else you would like to add?
